# Toll-Like Receptor (TLR) Signaling Enables Cyclic GMP-AMP Synthase (cGAS) Sensing of HIV-1 Infection in Macrophages

**DOI:** 10.1128/mBio.02817-21

**Published:** 2021-11-30

**Authors:** Mohammad Adnan Siddiqui, Masahiro Yamashita

**Affiliations:** a Aaron Diamond AIDS Research Center, Columbia University Vagelos College of Physicians and Surgeons, New York, New York, USA; UC Berkeley

**Keywords:** capsid, human immunodeficiency virus, innate immunity, interferons

## Abstract

HIV-1 replicates in cells that express a wide array of innate immune sensors and may do so simultaneously with other pathogens. How a coexisting innate immune stimulus influences the outcome of HIV-1 sensing, however, remains poorly understood. Here, we demonstrate that the activation of a second signaling pathway enables a cyclic GMP-AMP synthase (cGAS)-dependent type I interferon (IFN-I) response to HIV-1 infection. We used RNA sequencing to determine that HIV-1 alone induced few or no signs of an IFN-I response in THP-1 cells. In contrast, when supplemented with suboptimal levels of bacterial lipopolysaccharide (LPS), HIV-1 infection triggered the production of elevated levels of IFN-I and significant upregulation of interferon-stimulated genes. LPS-mediated enhancement of IFN-I production upon HIV-1 infection, which was observed in primary macrophages, was lost by blocking reverse transcription and with a hyperstable capsid, pointing to viral DNA being an essential immunostimulatory molecule. LPS also synergistically enhanced IFN-I production by cyclic GMP-AMP (cGAMP), a second messenger of cGAS. These observations suggest that the DNA sensor cGAS is responsible for a type I IFN response to HIV-1 in concert with LPS receptor Toll-like receptor 4 (TLR4). Small amounts of a TLR2 agonist also cooperate with HIV-1 to induce type I IFN production. These results demonstrate how subtle immunomodulatory activity renders HIV-1 capable of eliciting an IFN-I response through positive cross talk between cGAS and TLR sensing pathways.

## INTRODUCTION

The innate immune system detects invading pathogens, including viruses, and blocks their propagation before subsequent induction of adaptive immunity (1). Sensing of pathogen-associated molecular patterns (PAMPs) is mediated by a diverse set of pathogen recognition receptors (PRRs) and culminates in activation of an innate immune response (2). A major group of regulatory molecules involved in innate immune activation is type I interferons (IFN-I) (3), such as IFN-α and IFN-β, which establish an antiviral state by inducing expression of numerous IFN-stimulated genes (ISGs) (4). NF-κB, another key regulator, stimulates gene expression of proinflammatory cytokines and chemokines (5).

HIV-1, like many other viruses, produces virus-encoded molecules that can be sensed by the host innate immune system (6–10). However, HIV-1 is unique in that its replication cycle uses both RNA and DNA as genetic materials. As such, both viral DNA and RNA species can serve as PAMPs and be detected by cytosolic and endosomal PRRs, which include a broad array of receptors—RIG-I (11–13), MDA5 (14), DDX3 (15), cyclic GMP-AMP synthase (cGAS) (16, 17), IFI16 (18–20), PQBP1 (21), and various Toll-like receptors (TLR), including TLR3 (22, 23), TLR7 (22, 24–27), and TLR8 (25, 26, 28–30). Unintegrated viral DNA generated by incoming virus particles can be recognized as a foreign entity (16, 31), whereas RNA species, such as intron-containing genomic RNA (32, 33) and abortive RNA (15), can be detected by host sensors during both early and late phases. In addition to viral nucleic acids, virus-specific components can trigger an innate immune response. It has been reported that the viral capsid protein (CA) by itself (in the case of HIV-2), the CA domain of nascent Gag, and capsid lattice can be recognized by specific host factors: NONO, cyclophilin A, and TRIM5α (34–36). Additionally, HIV-1 virion assembly, if trapped by tetherin (also known as BST2), and HIV-1 entry (membrane fusion) elicit an innate immune response (37–40).

Previous research has firmly established that a number of molecules generated during HIV-1 infection have a capability to stimulate the innate immune system (10). In contrast, outcomes of innate sensing of HIV-1 infection *in vitro* vary widely across studies (41). A type I IFN response is a frequently utilized indicator of innate immune activation. Existing literature on the HIV-1-induced IFN-I response consists of two opposing observations; high levels of IFN-β gene induction or type I IFN production by HIV-1 infection was observed in some, but not all, labs (for example, see references 16 and 42). On the contrary, several studies have utilized induction of ISGs as a measure for HIV-1 sensing. A certain subset of ISGs were upregulated even without detectable levels of IFN-I upon HIV-1 infection of macrophages (13, 40, 43). It is likely that these markedly divergent outcomes of innate immune responses to HIV-1 infection are caused by context-dependent differences in a number of variables, such as cell types, virus strains, the size of inoculum, and mode of infection (44). For instance, infection through cell-to-cell transmission has been shown to be more efficient in inducing type I IFNs than infection with cell-free virus (45, 46).

Another key yet underappreciated aspect of HIV-1 sensing is cross talk between different innate immune pathways. Past studies have largely relied on purified virus stocks to avoid the confounding effects of any coexisting immunostimulatory molecules, which could generate “noises” in assay readouts or interact with HIV-mediated signaling pathways (47). However, HIV-1 target cells, such as macrophages, express a wide range of innate immune sensors. In pathogenic HIV infections, microbial products translocate from the gastrointestinal tract into the systemic circulation (48–50). Hence, it is possible that HIV-1 is sensed in the presence of other pathogens or immunostimulatory signals. Importantly, recent work by Johnson et al. showed that HIV-induced cGAS activation primes virus-infected dendritic cells for elevated innate immune activation by independent second signals (51). However, it is currently unclear how a coexisting, distinct signal would affect cGAS sensing of HIV-1 infection. We initiated this work upon our serendipitous discovery that has helped us address this question. In this work, using an unbiased transcriptome approach, we show that HIV-1 infection by itself displays little or no activity to induce a type I IFN response in THP-1 cells. In contrast, coadministration of small amounts of TLR agonists during HIV-1 infection induces a strong type I IFN response, which depends on viral DNA synthesis and the “correct” capsid. These results reveal how a subtle, coexisting signal enables HIV-1 to fully stimulate the cGAS-stimulator of interferon genes (STING) signaling pathway.

## RESULTS

### Virus preparation methods influence HIV-induced production of type I IFNs.

**FIG 1:**
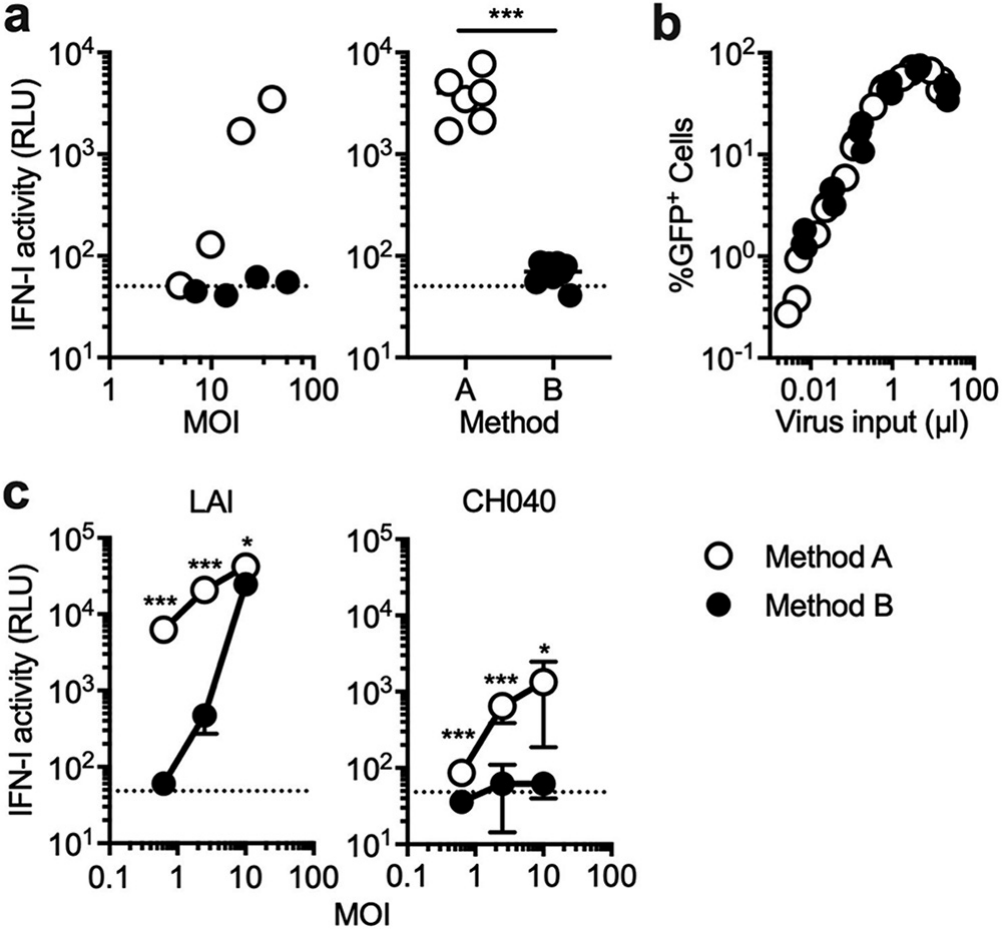
HIV-1-induced IFN-I production differs between plasmid purification protocols. Virus stocks were prepared using two different sources of plasmid DNA (method A and B). (a and c) THP-1 cells or primary monocyte-derived macrophages (MDMs) were infected with VSV-G-pseudotyped, GFP-encoding reporter HIV-1 across various multiplicities of infection (MOIs). A fraction of the culture supernatant harvested 1 day postinfection (dpi) was used to assess the amount of IFN-I using a reporter cell line that expresses luciferase under the control of the ISRE promoter. IFN-I activity is represented by luciferase activity and shown as relative light units (RLU). (a) THP-1 cells were infected with different amounts of VSV-G-pseudotyped GFP reporter viruses. The results shown in the left panel represent those of three independent experiments. The difference in the levels of IFN-I between two conditions was analyzed for statistical significance (right panel) using the two-tailed, unpaired Student’s *t* test. Samples of the cells that were infected with MOIs of less than 10 were excluded for analysis, as this level of infectivity was incapable of inducing IFN-I production even with virus prepared with method A. (b) Infectivity of virus stocks prepared with two different methods was compared by infecting THP-1 cells with various amounts of virus inputs. At 2 dpi, virus-infected cells were fixed and examined for the number of cells that express GFP encoded by reporter virus. The graph was compiled by using the results from three independent experiments. (c) MDMs were infected with VSV-G-pseudotyped GFP reporter viruses encoding CA from LAI or CH040 with increasing amounts of viral input. The results from two independent experiments were combined, and the average of triplicate samples was used to plot the graph, with error bars denoting the standard deviation (SD). Differences in IFN-I activity between two conditions were examined for statistical significance using the two-tailed, unpaired Student’s *t* test. Horizontal dashed lines represent baselines, which are the average of RLU values for mock-infected cells. ***, *P* < 0.001; *, *P* < 0.05.

In our previous work, we observed that HIV-1 induced high levels of IFN-I production in a cGAS-dependent manner when large sizes of the inoculum were used to infect monocytic THP-1 cells and macrophages (52). However, we found that this ability was almost entirely lost when a technical modification was introduced in a procedure to extract plasmid DNA for generating virus stocks via transfection (Fig. 1). In this work, we studied the underlying mechanisms of this batch-to-batch variation in the hope of gaining new insights into the regulation of HIV-1 innate sensing in myeloid cells.

Plasmid DNA stocks were prepared using two commercial kits with slightly differing protocols, a PureYield Plasmid Midiprep system kit and a NucleoBond PC 10000 EF Giga kit. The protocol of the former kit largely relies on column purification, while the latter kit similarly uses column purification but requires an additional step of centrifugation to precipitate plasmid DNA. In this paper, a protocol to generate virus stocks using the former kit is called method A, while the one using the latter kit is called method B. To measure the amount of IFN-I in the supernatant from virus-infected THP-1 cells, we quantified their biological activity on a reporter cell line (53). These two types of viruses exhibited drastically different activity to induce IFN-I in THP-1 cells (Fig. 1). The wild-type (WT) virus prepared with method A induced robust IFN-I production, whereas the counterpart prepared with method B elicited significantly lower levels of IFN-I in THP-1 cells (Fig. 1a). Note that there were no discernible differences in infectivity between these virus stocks (Fig. 1b), suggesting that the two opposing phenotypes of IFN-I production are not caused by differences in the amount of virus-related PAMPs (e.g., newly generated viral DNA).

We extended this finding to primary monocyte-derived macrophages (MDMs), a physiologically relevant cell type. The amount of IFN-I secreted after infection of MDMs with the WT virus was significantly higher when the virus stock was prepared using method A rather than method B (Fig. 1c). The difference in the quantity of IFN-I between these two methods was more prominent at low multiplicities of infection (MOI) than at a high MOI, as virus prepared with method B induced IFN-I at a high MOI (Fig. 1c). The viral capsid controls the exposure of viral nucleic acids to innate sensors and thereby regulates the outcome of an innate immune response upon HIV-1 infection (54, 55). Our previous work reported that CH040, a strain of HIV-1, has a distinct capsid that conferred the ability to evade viral DNA sensing by cGAS (52). Consistently, the capsid of CH040 significantly weakened the ability of HIV-1 to induce IFN-I production by MDMs in both preparation methods compared to the wild-type virus carrying CA from the LAI strain (Fig. 1c).

The difference in the amount of IFN-I induced between these two virus stocks suggests that virus stocks prepared with method A contain a component that enables myeloid cells to mount robust IFN-I production upon HIV-1 infection. However, our findings also show that neither such putative component nor HIV-1 infection by itself is sufficient for inducing high levels of IFN-I production in these cell types. In other words, the combination of these two signals is required for the observed type I IFN response.

### Different methods of virus preparation cause discordant gene expression patterns for the IFN signaling pathway.

**FIG 2:**
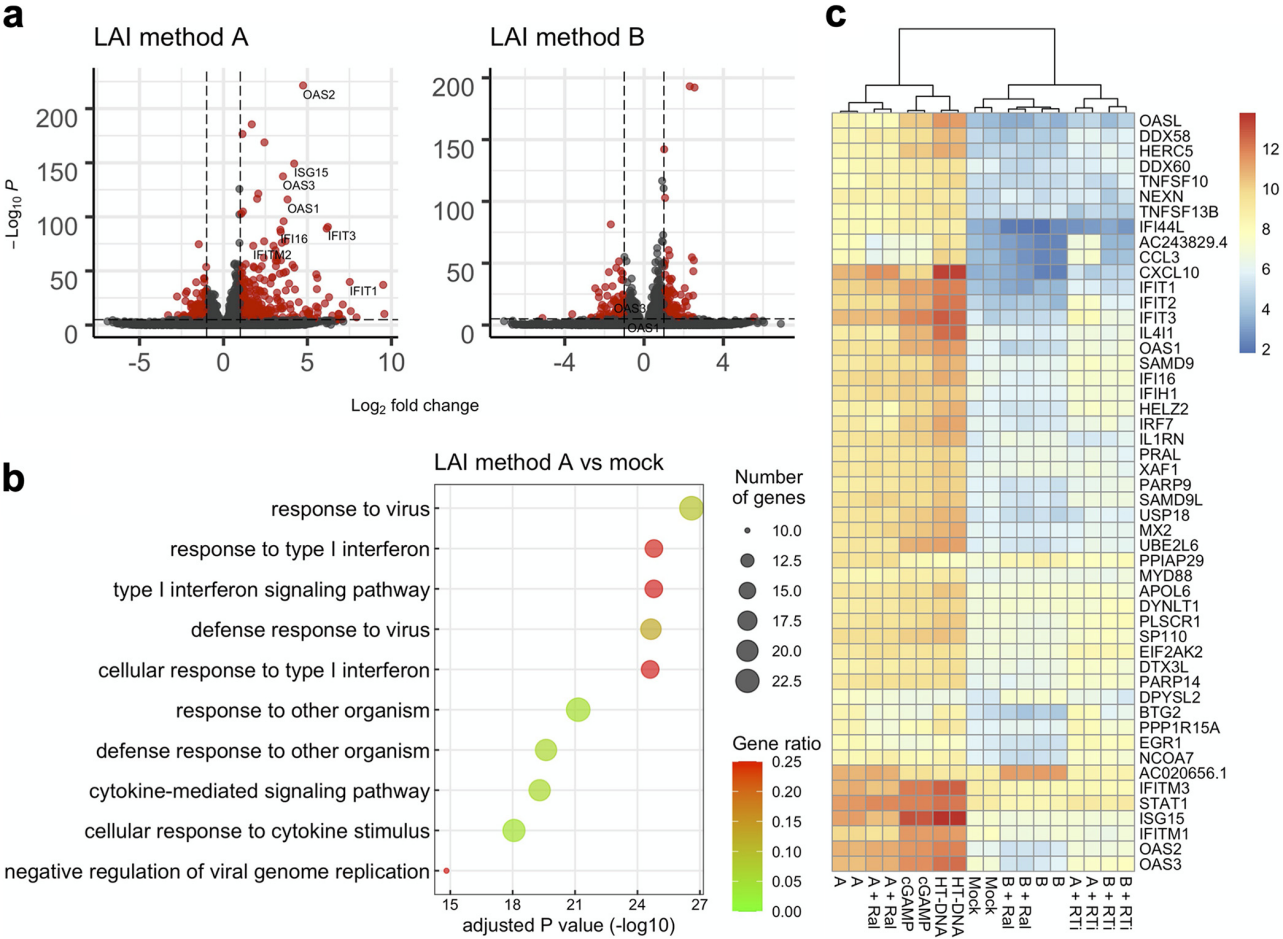
A gene expression signature of type I IFN signaling depends on plasmid purification protocols and viral DNA synthesis. THP-1 cells were either infected with HIV-1 under different conditions or treated with cGAS stimulatory molecules (cGAMP and HT-DNA). Total RNA used for construction of RNA-sequencing libraries was extracted from cells harvested at 16 h after infection, except for those treated with cGAMP or HT-DNA, which were harvested at 2 h after treatment. (a) Volcano plots depict gene expression differences as fold differences (log_2_ transformed) between mock-infected cells and virus-infected cells. A representative set of ISGs is labeled. Horizontal dashed lines correspond to an arbitrary cutoff *P* value of 10^−6^. Vertical lines correspond to a log_2_ fold change of ± 2. Genes that satisfy these cutoff values are shown in red, while those that do not are shown in black. (b) Shown are the top 10 Gene Ontology (GO) terms enriched in the sample prepared from THP-1 cells infected with the WT virus prepared using method A. All these terms belong to a GO aspect called “biological process.” Genes with a log_2_ fold change of greater than 2 were ranked based on adjusted *P* values before the top 50 upregulated genes were selected and subjected to GO analysis. Dot sizes indicate the number of genes associated with a corresponding GO term, whereas dot colors indicate the proportion of enriched genes in all the genes that belong to a given GO term. (c) The heat map depicts gene expression levels across different samples and was generated using normalized read counts. The top 50 upregulated genes in the sample “A” (i.e., LAI virus prepared with method A) compared to the mock-infected samples are shown. These upregulated genes were selected with the following criteria: adjusted *P* values of <0.01, log_2_ fold change of >2. Ral, raltegravir; RTi, reverse transcription inhibitors (100 μM lamivudine [3TC], 200 μM AZT, 50 μM nevirapine).

Previous work has shown that HIV-1 induces a certain set of ISGs without causing production of detectable levels of type I IFNs (13, 40, 43, 56–58). To test if this were the case for the batch of virus that failed to cause IFN-I production, we used RNA sequencing to perform transcriptome analysis of THP-1 cells infected with HIV-1 under a variety of experimental conditions. THP-1 cells were chosen for transcriptome analysis because we reasoned that an almost complete lack of IFN-I activity by virus prepared with method B would allow us to identify a determinant for the observed difference between these two virus preparations. As expected, infection with the LAI virus, which was prepared using method A and induced high levels of IFN-I in the supernatant, upregulated the expression of many ISGs, such as IFIT1 and OAS1 (Fig. 2a). Gene Ontology (GO) analysis showed that significantly upregulated genes in cells infected with this batch of virus were indeed overrepresented in GO terms related to the type I IFN response (Fig. 2b). In marked contrast, the LAI virus prepared with method B, which failed to induce robust IFN-I production (Fig. 1), did not upregulate the same set of ISGs at equivalent levels (Fig. 2a).

Multiple studies showed that the principal molecule that mediates HIV-1 sensing in THP-1 cells is cGAS, an innate sensing molecule that recognizes DNA (16, 21, 52, 59, 60). Consistently, treatment with a cocktail of reverse transcriptase inhibitors, but not an integrase inhibitor (raltegravir), drastically blocked an increase in expression of the almost entire set of ISGs induced upon infection with the LAI virus prepared with method A (Fig. 2c). These treatments did not overtly affect the inability of the LAI virus prepared with method B to activate type I IFN signaling. Taken together, the transcriptome analysis demonstrated that a lack of the IFN-I response by a certain batch of the virus was not merely due to the detection limit of the bioassay. Furthermore, our analysis supports the idea that incoming HIV-1 particles effectively evade innate sensing. On the contrary, our data also showed that when a type I IFN response was induced by a certain batch of HIV-1, it required viral DNA synthesis but not integration. In addition, cGAS depletion by short hairpin RNA (shRNA) drastically decreased the level of type I IFNs induced by the LAI virus prepared with method A (Fig. S1). The specificity of the effects of cGAS depletion was validated by a similar decrease in the amount of produced type I IFNs upon transfection with herring testis (HT) DNA, but not by transfection with cyclic GMP-AMP (cGAMP), a second messenger downstream of cGAS sensing of DNA, or with poly(I·C), synthetic double-stranded RNA that can stimulate IFN induction through the TLR3 receptor. These results are consistent with the previous observation that cGAS is the key DNA sensor for HIV-1 in THP-1 cells (16, 21, 52, 59, 60).

10.1128/mBio.02817-21.1 FIG S1 cGAS is required for induction of type I IFNs by a certain batch of HIV-1. THP-1 cells were transduced with lentiviruses that do not express shRNA (empty) or express shRNA targeting cGAS. (a) mRNA levels of cGAS were measured using qRT-PCR. The relative amount of cGAS mRNA was normalized to the mRNA level of GAPDH in each sample, and the fold differences of the transduced samples were compared with the nontransduced sample. Transduction was performed in triplicate. The results are shown as the mean with error bars denoting the SEM. The two-tailed, unpaired Student’s *t* test was used to examine the difference between cGAS-depleted cells and two control samples. (b) THP-1 cells were infected with VSV-G-pseudotyped HIV-1 GFP reporter virus prepared with method A or transfected with HT-DNA (2 μg per mL), cGAMP (20 μg per mL), or poly(I:C) (2 μg per mL). IFN-I production in the culture supernatant was measured at 24 h after treatment using the HEK293 cell-based reporter cells. Two independent experiments were performed in three biological replicates. The results are shown as the mean with error bars denoting SEM. *P* values were calculated after ANOVA using Tukey’s multiple-comparison test. ****, *P* < 0.0001. Download FIG S1, TIF file, 0.5 MB. Copyright © 2021 Siddiqui and Yamashita. 2021 Siddiqui and Yamashita. https://creativecommons.org/licenses/by/4.0/ This content is distributed under the terms of the Creative Commons Attribution 4.0 International license.

### Proinflammatory responses are triggered by viruses that activate type I IFN signaling.

**FIG 3:**
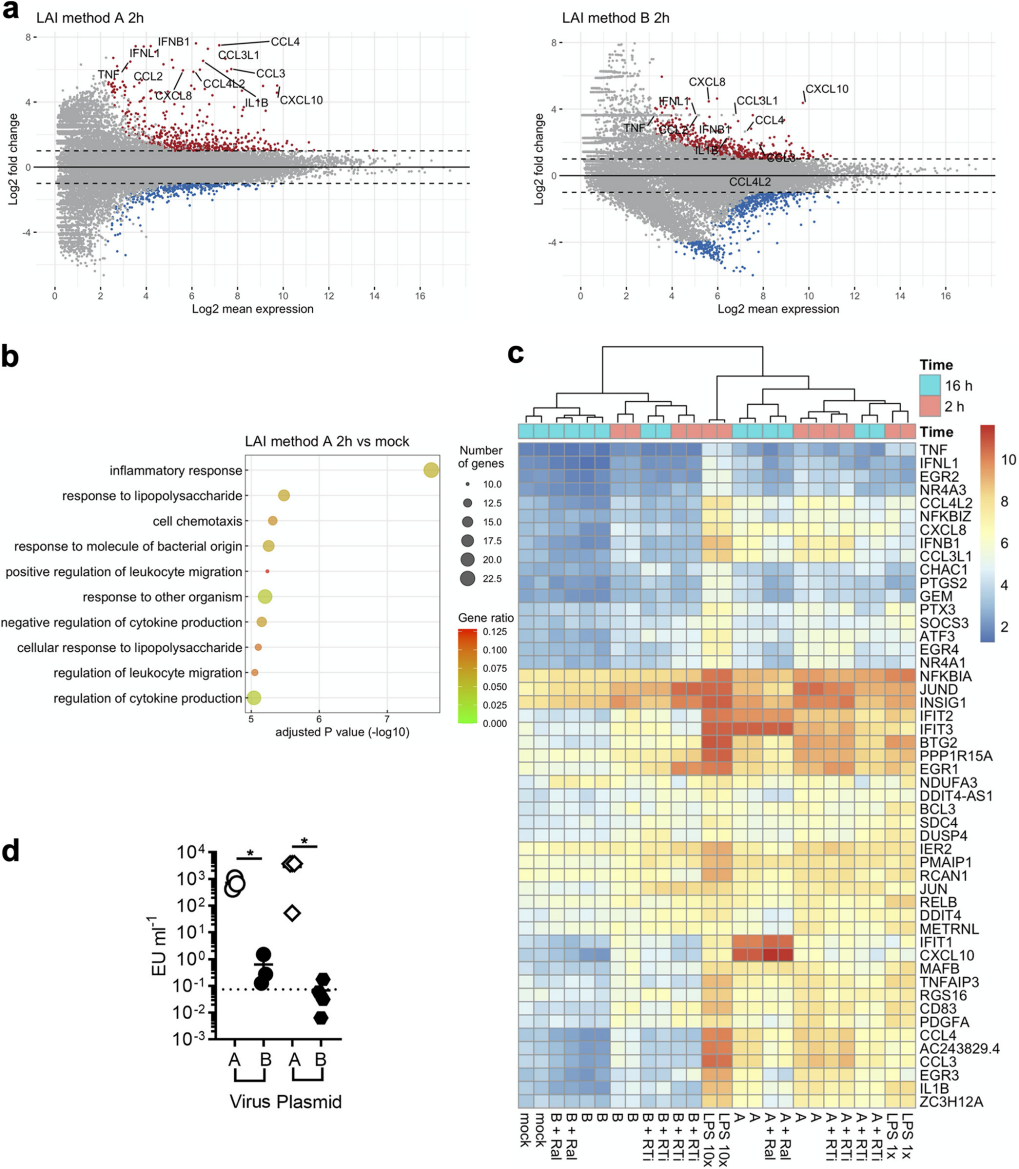
Virus stocks that activate IFN signaling upregulate LPS-induced genes and contain elevated levels of LPS. (a) Gene expression profiles of two samples of the indicated conditions (cells harvested at 2 h postinfection) were compared to those of mock-infected cells. In these 2-dimensional scatterplots, log_2_ fold changes between two conditions are used on the *y* axis, while the log_2_-transformed mean of normalized expression counts is plotted on the *x* axis. Shown in red and blue are genes that have a log_2_ fold change that is greater than 2 or less than –2 and have adjusted *P* values less than 0.05. Red dots are upregulated genes, while blue dots are downregulated genes. Representative proinflammatory genes are labeled. (b) Top 10 GO terms enriched in the sample from THP-1 cells with virus stock A and harvested at 2 h postinfection. See the legend of Fig. 2 for details. (c) Transcriptional profiles of THP-1 cells infected with HIV-1 under various conditions were examined based on significantly upregulated genes by LPS (10×, 200 μg/mL; 1×, 20 μg/mL). The top 50 genes with largest variance among LPS-induced genes across these samples were chosen to draw the heat map using a normalized read count. Abbreviations are the same as those used in Fig. 2. Time points of sample harvesting are shown by colored boxes on the top of the heat map. Samples collected at 2 and 16 h postinfection are shown in magenta and cyan, respectively. (d) The amount of LPS in plasmid and virus stocks was measured with a standard assay using amebocyte lysates. Data values were generated using four different plasmid stocks and three virus stocks. Data sets were analyzed using the two-tailed, unpaired Student’s *t* test. A horizontal dashed line represents the limit of detection, which is the lowest value within the linear range of the standard curve that falls in the linear range. EU, endotoxin unit; *, *P* < 0.05.

In our experimental setting, activation of type I IFN signaling by HIV-1 infection was determined, in part, by the method for plasmid DNA purification. One possible scenario is that virus stocks prepared using method A contain a constituent that is required for IFN-I production upon HIV-1 infection. Note that such a constituent is not sufficient for stimulating IFN-I production by itself, as blocking reverse transcription effectively prevented an IFN-I response (Fig. 2c). We reasoned that such a constituent is of bacterial origin and therefore focused our attention on lipopolysaccharide (LPS), a membrane component of Gram-negative bacteria that can induce a proinflammatory response. Consistently, we found that even at an early time point (2 h postinfection), virus prepared with method A induced elevated gene expression of a variety of proinflammatory cytokines and chemokines compared to virus prepared with method B (Fig. 3a). GO analysis indeed revealed that highly upregulated genes by virus prepared with method A are related to the inflammatory response as well as LPS (Fig. 3b). Expression profiling based on a set of LPS-induced genes revealed two distinct clusters in a heat map, which perfectly match the methods of plasmid preparation (Fig. 3c). Specifically, all the samples that were infected with virus stocks prepared using method A displayed a pattern indistinguishable from those treated with a smaller amount of LPS (LPS 1× in Fig. 3c). In marked contrast, infection with virus stocks prepared with method B resulted in upregulation of a more limited set of the LPS-induced genes; this upregulation was also to a lesser degree than observed after infection with those prepared with method A. Importantly, upregulation of these genes was observed at 2 h postinfection, a time point at which fewer ISGs were induced in the same set of samples (data not shown) and not blocked by reverse transcriptase inhibitors. This finding suggests that the observed proinflammatory response is not the consequence of cGAS-dependent HIV-1 sensing. Overall, these results support the idea that a constituent specific to virus stocks prepared with method A drives a proinflammatory response, which resembles the one induced by LPS.

### High levels of endotoxin are present in HIV-1 stocks that induced IFN-I production.

We next measured the quantity of LPS in both plasmid DNA and virus stocks using a commercial kit (Fig. 3d). Virus stocks generated with method A contained an elevated level of LPS compared to virus stocks generated with method B (Fig. 3d). Similarly, high levels of LPS were detected in plasmid DNA stocks generated using method A, in contrast to the smaller amounts of LPS in plasmid DNA stocks generated using method B (Fig. 3d). Therefore, the amount of LPS present in plasmid DNA stocks was correlated with the amount of LPS in virus stocks and, more importantly, with the ability of these virus stocks to induce high levels of IFN-I.

### Loss of LPS attenuates HIV-mediated induction of IFN-I.

**FIG 4:**
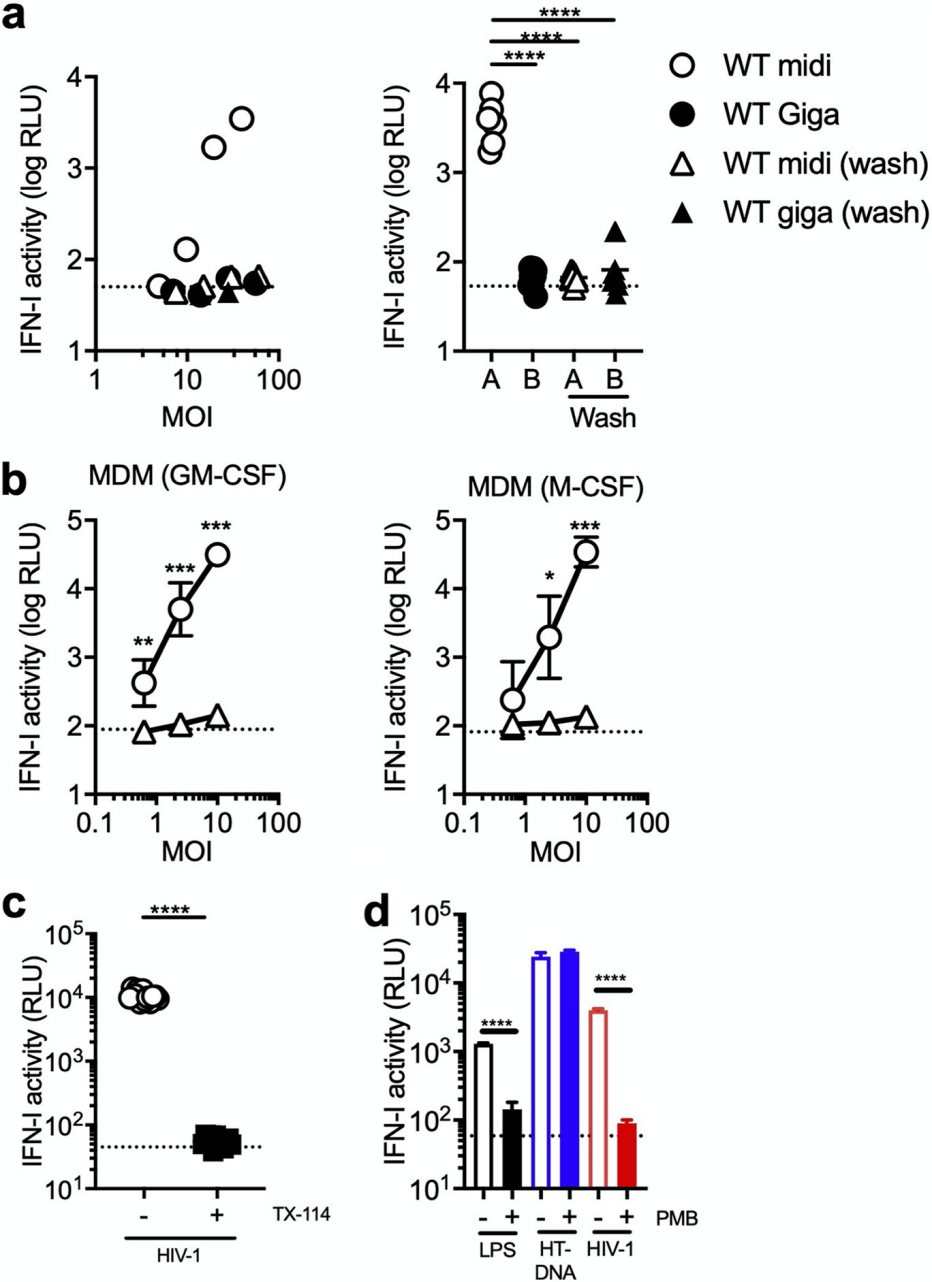
Loss of LPS abrogates a type I IFN response to HIV-1 infection. (a and b). Virus stocks of the WT HIV-1 strain were prepared using method A or B. Corresponding stocks were also generated by replacing culture medium at 1 day posttransfection (“+ wash”). They were used to infect THP-1 cells (a) or MDM (b). (a) One representative result (*n* = 3) is shown in the left panel. The right panel was generated using the results of samples infected at MOIs higher than 10 because no noticeable IFN production was observed at MOIs lower than 10. (b) Primary MDMs were prepared using CD14^+^ cells in PBMCs from two donors. MDMs were differentiated with either GM-CSF or M-CSF. Two different virus stocks (method A versus method A + wash) were used to infect MDMs across different MOIs. The average values of results from three independent experiments were used to plot the graph, with error bars denoting the standard error of the mean (SEM). The average values of IFN-I activity of mock-infected samples are shown as horizontal dashed lines. (c) THP-1 cells were infected with virus stocks of the GFP reporter virus for the WT HIV-1 strain that were generated using method A. A single dose of virus input was selected because it was predetermined to induce IFN-I induction in THP-1 cells. Plasmid DNA used for generating virus stocks was treated with or without Triton X-114 (TX-114). The results displayed here are the average of those from two independent experiments. Dashed lines in each panel denote the average of IFN-I activity for mock-infected samples. (d) THP-1 cells were either transfected or infected in the presence or absence of polymyxin B (PMB), an LPS-signaling inhibitor (100 μg/mL). Transfection was performed with LPS at 10 μg/mL or herring testes DNA (HT-DNA) at 0.2 μg/mL. Infection was performed using virus stocks for the WT HIV-1 strain that were prepared with method A. The result shown was compiled from two independent experiments, with error bars denoting the SEM. In all the panels, the amount of IFN-I secreted in the supernatant harvested 1 day after transfection or infection was quantitated as described in the legend of Fig. 1. Statistical significance shown in this figure was analyzed using the two-tailed, unpaired Student’s *t* test. RLU, relative light units. ****, *P* < 0.0001; ***, *P* < 0.001; **, *P* < 0.01; *, *P* < 0.05.

Virus stocks used in these experiments were purified with ultracentrifugation. It is likely that LPS remained in the culture supernatant after transfection and was copelleted with virus particles. Introduction of the washout step during virus production drastically reduced IFN-I induction elicited by HIV-1 infection of THP-1 cells (Fig. 4a). These observations were largely reproducible in MDMs. We tested these viruses using MDMs prepared with granulocyte-macrophage colony-stimulating factor (GM-CSF) or M-CSF, as it was reported that these two types of MDMs display slightly different patterns of gene expression (61). However, regardless of MDM preparation procedures, addition of the washout process during the virus preparation step almost completely eliminated the ability of virus prepared with method A to induce a type I IFN response in two preparations of MDMs (Fig. 4b).

The amount of LPS in plasmid DNA stocks can be reduced by treatment with Triton X-114 (62). Virus stocks generated using Triton X-114-treated DNA stock were significantly less effective in inducing IFN-I than those generated using untreated plasmid DNA (Fig. 4c), even though both viruses resulted in similar levels of infectivity (data not shown). Polymyxin B (PMB) is a cyclic peptide that suppresses the biological activities of LPS by forming complexes with lipid A, a negatively charged component of LPS (63). Consistent with this property, PMB treatment of THP-1 cells blocked IFN-I production induced by LPS (Fig. 4d). The effect of PMB appeared to be specific for LPS, as PMB did not show an inhibitory effect on DNA sensor-dependent IFN-I induction produced upon transfection of herring testes DNA (Fig. 4d). Importantly, PMB treatment reduced the level of IFN-I induction by THP-1 cells infected with HIV-1 prepared with method A (Fig. 4d). Thus, the ability of HIV-1 to induce high levels of type I IFN in THP-1 cells and macrophages was lost upon various measures to remove or inactivate LPS.

A previous report showed that the G protein of vesicular stomatitis virus (VSV) forms a tubulovesicular structure that can stimulate type I IFN induction via TLR9 in plasmacytoid dendritic cells (pDCs) (64). To dissect the role of the VSV-G protein in the observed type I IFN response in our experimental system, we generated green fluorescent protein (GFP) reporter viruses that were pseudotyped with HIV-1 Env from the CCR5-tropic YU2 strain using method A. Infection of MDMs with this virus stock pseudotyped with HIV-1 Env induced type I IFN production (Fig. S2a). Similar to the experiments described above (Fig. 4a), introduction of the same washout process during virus production impaired the ability of the virus stock pseudotyped with HIV-1 Env to stimulate type I IFN induction (Fig. S2a). In addition, replication-competent virus carrying the *env* gene from the CCR5-tropic BaL strain did not induce type I IFNs when the transfection procedure included a step to remove the transfection input (Fig. S2b) These results indicate that the observed induction of type I IFNs upon HIV-1 infection is caused independently from the VSV-G protein.

10.1128/mBio.02817-21.2 FIG S2 Effects of HIV-1 Env on the ability of HIV-1 to elicit production of type I IFNs. (a) GFP-reporter virus pseudotyped with HIV-1 Env from the CCR5-tropic YU2 strain was generated using method A with or without a washing process. Different amounts of virus input were used to infect MDMs from two donors in three different experiments (*n* = 6). The amount of IFN-I in the supernatant harvested at 1 dpi was quantified as described in the legend to Fig. 1. The results are shown as the mean with error bars denoting the SEM. The two-tailed, unpaired Student’s *t* test was used to examine the difference between two conditions. **, *P* < 0.01; ns, not significant. (b) Replication-competent virus encoding the BaL Env carrying GFP reporter was generated by transfection of 293T cells followed by a washout process. This virus stock was titrated on C8166 cells expressing CCR5 along with VSV-G pseudotyped virus stocks prepared by method A or method B. Increasing amounts of virus input were used to infect MDMs from two donors in two independent experiments (*n* = 4) Error bars show the standard deviation. The differences in the amounts of IFN induced by virus inputs that range from MOIs of 0.5 to 4 were compared using Welch’s unpaired *t* test. ****, *P* < 0.0001. Download FIG S2, TIF file, 0.3 MB. Copyright © 2021 Siddiqui and Yamashita. 2021 Siddiqui and Yamashita. https://creativecommons.org/licenses/by/4.0/ This content is distributed under the terms of the Creative Commons Attribution 4.0 International license.

### LPS cooperates with HIV-1 to induce type I IFN production.

**FIG 5:**
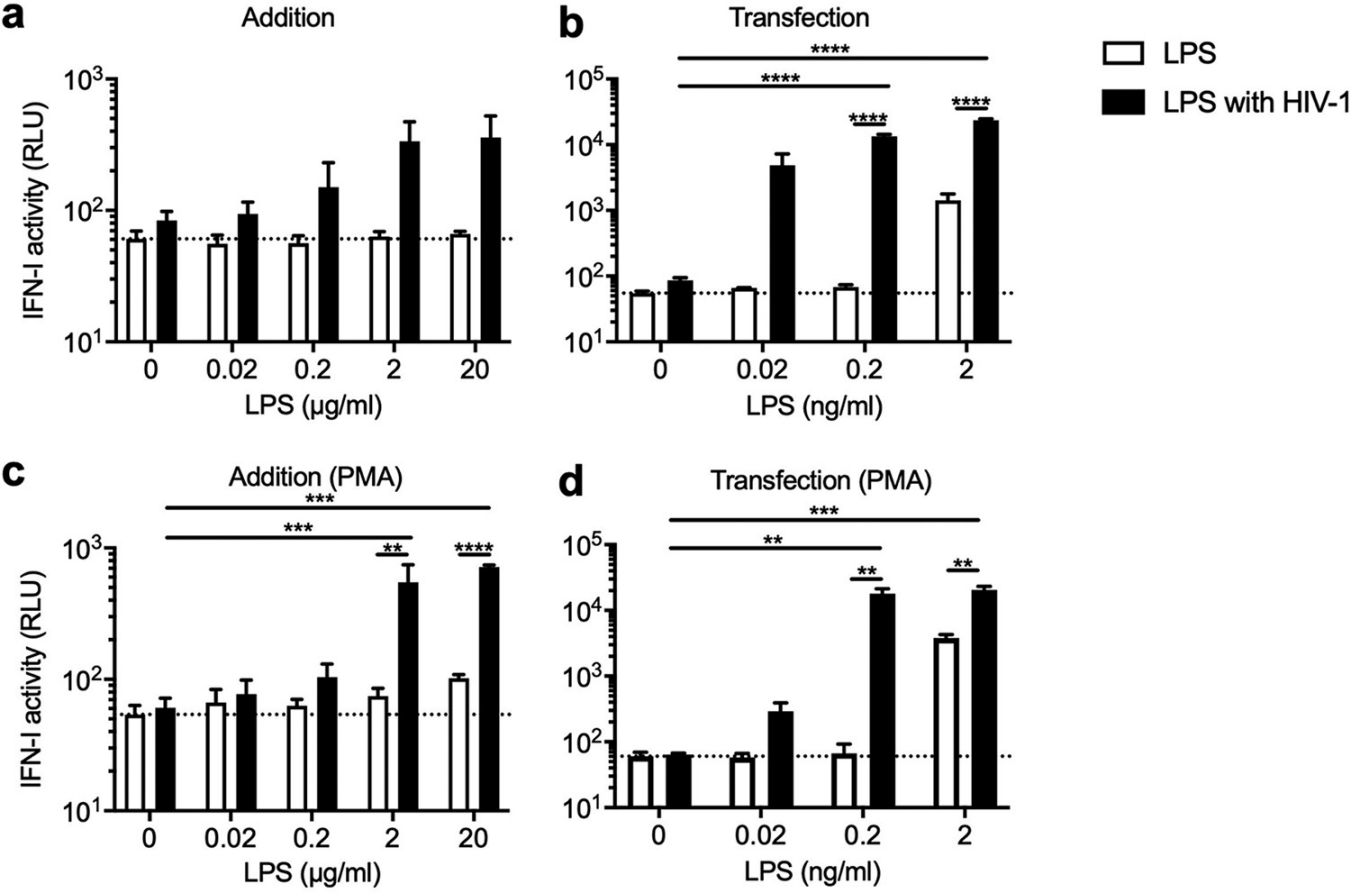
HIV-1 and LPS synergize to elicit a type I IFN response. A fixed amount of GFP reporter virus that does not induce high levels of IFN-I by itself was used to infect untreated (a and b) or PMA-treated (c and d) THP-1 cells, which were subsequently treated with increasing amounts of LPS. These suboptimal quantities of LPS alone (white bars) did not induce detectable levels of IFN-I, except for one condition (transfection with 2 ng/mL of LPS). LPS was directly supplied to the cells in culture (a and c) or transfected (b and d). The amount of IFN-I in the supernatant harvested at 1 dpi was quantified as described in the legend of Fig. 1. The results are shown as the mean with SEM (*n* = 3). The averages of type I IFN activity of mock-infected samples are indicated as horizontal dashed lines in each graph. *P* values were calculated after ANOVA using Tukey’s multiple-comparison test. ****, *P* < 0.0001; ***, *P* < 0.001; **, *P* < 0.01.

We next asked whether LPS can confer the ability to induce a high level of IFN-I in HIV-1 stocks that lack the same ability. To test this, we treated THP-1 cells with LPS before infection with reporter virus of the LAI strain of HIV-1 prepared with method B, which did not induce detectable levels of IFN-I (Fig. 1). The amount of LPS used in this experiment was determined *a priori* so that LPS treatment alone did not induce production of high levels of IFN-I. Furthermore, cells were also treated with LPS transfection, which can stimulate a TLR4-independent intracellular inflammasome complex (65). In the experiments for LPS transfection, the amount of LPS needed was lower than the amounts used in the addition experiment by approximately 1,000-fold (Fig. 5). Treatment of THP-1 with LPS at these suboptimal levels did not cause a release of detectable IFN-I activity, except for samples transfected with 2 ng per mL of LPS (Fig. 5b). Infection of THP-1 cells with virus prepared using method B alone failed to induce elevated levels of IFN-I production, although IFN-I activity measured by luciferase activity was slightly higher than the baseline (Fig. 5a; see two values at 0 μg per mL of LPS). Remarkably, addition of increasing amounts of LPS caused production of increasing levels of IFN-I activity (Fig. 5a). Transfection of LPS gave rise to a similar but more pronounced pattern; >100-fold increases in the level of IFN-I were observed when cells were transfected with LPS along with HIV-1 infection (Fig. 5b). Note that the observed induction of IFN-I was reduced to a level similar to those of control cells by treatment with a cocktail of reverse transcriptase inhibitors (Fig. S3), suggesting that viral DNA sensing is essential for induction of IFN-I by the cooperative action of LPS and HIV-1. These observations were made with undifferentiated THP-1 cells but reproduced with differentiated THP-1 cells, which acquired a macrophage-like phenotype upon treatment with phorbol 12-myristate 13-acetate (Fig. 5c and d).

10.1128/mBio.02817-21.3 FIG S3 Viral DNA synthesis is required for the synergy between HIV-1 and LPS to induce a type I IFN response. A fixed amount of GFP reporter virus that does not induce production of detectable levels of type I IFNs by itself was used to infect THP-1 cells that were treated with increasing but suboptimal amounts of LPS by transfection in the presence or absence of a cocktail of reverse transcriptase inhibitors (RTi), including AZT (10 μM), 3TC (20 μM), and nevirapine (5 μM). These quantities of LPS alone (white bars) did not induce high levels of IFN-I. The amount of IFN-I in the supernatant harvested at 1 dpi was quantified as described in the legend to Fig. 1. The results are shown as the mean of six replicates in two independent experiments, with error bars denoting the SEM. The average of type I IFN activity of mock-infected samples is indicated as a horizontal dashed line. *P* values were calculated after ANOVA using Tukey’s multiple-comparison test. *, *P* < 0.05. Download FIG S3, TIF file, 0.4 MB. Copyright © 2021 Siddiqui and Yamashita. 2021 Siddiqui and Yamashita. https://creativecommons.org/licenses/by/4.0/ This content is distributed under the terms of the Creative Commons Attribution 4.0 International license.

### LPS enhances IFN-I production by cGAMP.

**FIG 6:**
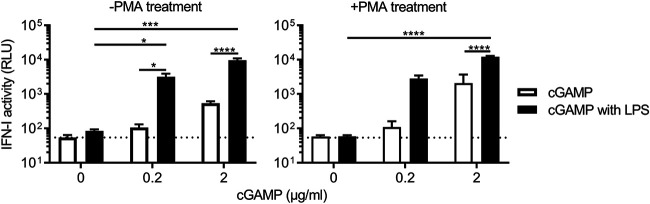
LPS cooperate with cGAMP to increase production of type I IFNs. THP-1 cells that were either left untreated or treated with PMA were transfected with LPS (2 μg/mL) alone, cGAMP alone, or both together. Experiments were performed in six replicates across four independent experiments. One data set of results for untreated cells was removed from analysis because LPS transfection alone induced a high level of IFNs. See the legend of Fig. 1 for the details of the method for measurement of type I IFN. Error bars denote the SEM. A dashed line indicates the assay baseline, which is the average of type I IFN activity of mock-treated samples. The statistical significance of differences between each condition was assessed using ANOVA. *P* values for pairwise comparison were calculated using Tukey’s multiple-comparison test. ****, *P* < 0.0001; ***, *P* < 0.001; *, *P* < 0.05.

These results support the idea of cross talk between LPS and cGAS signaling pathways. We next examined a step that can be facilitated by LPS using cGAMP, a second messenger in the cGAS signaling pathway (66). cGAMP is produced upon DNA binding to cGAS and hence enables us to study downstream events involving STING-dependent signal transduction. The concentrations of 2′3′-cGAMP used induce suboptimal levels of type I IFNs in our bioassay (white bars in Fig. 6), whereas the LPS concentration used in this experiment induces almost undetectable levels of type I IFNs (black bars with 0 μg/mL of cGAMP in Fig. 6). However, when the two molecules were mixed together prior to transfection of untreated and phorbol myristate acetate (PMA)-treated cells, they allowed for a drastic increase in the level of IFN-I (Fig. 6). Since cGAMP is generated upon the binding of cGAS to DNA, these results suggest that LPS can facilitate a step downstream of cGAMP production to enhance IFN-I production.

### TLR2 agonist enables IFN-I production upon HIV-1 infection of THP-1 cells.

**FIG 7:**
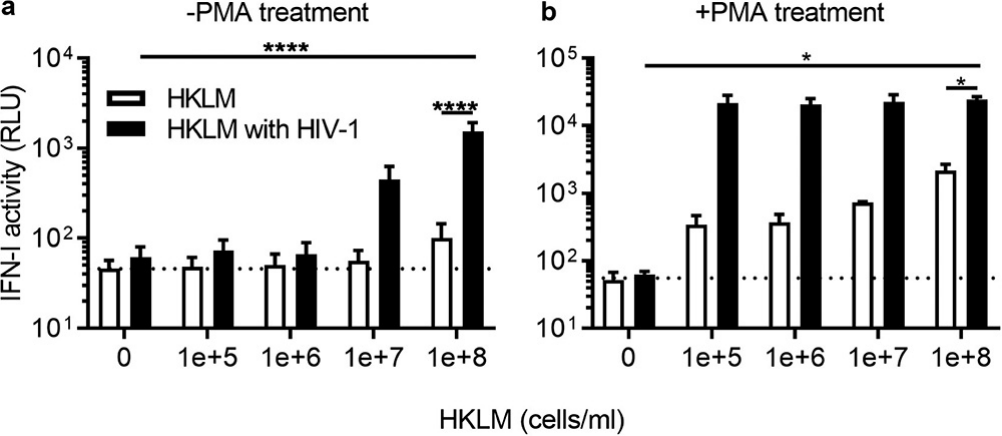
TLR2 activation by HKLM unmasks the ability of HIV-1 to stimulate IFN signaling. HKLM (heat-killed preparation of Listeria monocytogenes) was added on the day of infection to THP-1 cells that were either pretreated with or without PMA. HKLM-treated cells were then infected with a single dose of GFP-reporter virus that by itself does not induce significant levels of type I IFN (see the black bars with 0 cell per mL of HKLM). The amount of type I IFN in the supernatant at 1 dpi was quantified as described in the legend of Fig. 1. The results shown here are the mean of type I IFN activity obtained in three independent experiments. Error bars denote the SEM. The average of IFN-I activity for mock-treated samples is shown as a horizontal dashed line. Statistical significance for the difference shown in the figure was calculated using ANOVA, followed by Tukey’s multiple-comparison test. ****, *P* < 0.0001; *, *P* < 0.05.

LPS, along with a variety of molecular patterns, such as nucleic acids of bacterial and viral origin, are sensed by TLRs (67). LPS signaling is initiated by its binding to TLR4 and subsequent activation of innate immune responses through the transcription factors NF-κB and interferon regulatory factor 3 (IRF3). Given that other TLRs are similarly capable of eliciting an innate immune response, we hypothesized that a different TLR agonist can also enhance type I IFN production stimulated by HIV-1. Here, we used a TLR2 agonist called HKLM. TLR2 recognizes major PAMPs of Gram-positive bacteria, including lipoteichoic acid, a key component of cell walls (68). HKLM is a freeze-dried, heat-killed preparation of the Gram-positive bacterium Listeria monocytogenes. HKLM was titrated on THP-1 cells at different concentrations to identify concentrations that cause undetectable or low IFN-I production (69, 70) (Fig. 7). Consistent with our idea, we found that addition of HKLM conferred the ability of virus prepared with method B to induce a markedly increased level of IFN-I compared to that of each stimulation alone (Fig. 7). Taken together, these results demonstrate that suboptimal levels of TLR ligands have drastic impacts on the consequence of HIV-1 sensing in THP-1 and macrophages.

### Capsids deficient for CPSF6 binding do not render HIV-1 capable of inducing a type I IFN response in THP-1 cells.

**FIG 8:**
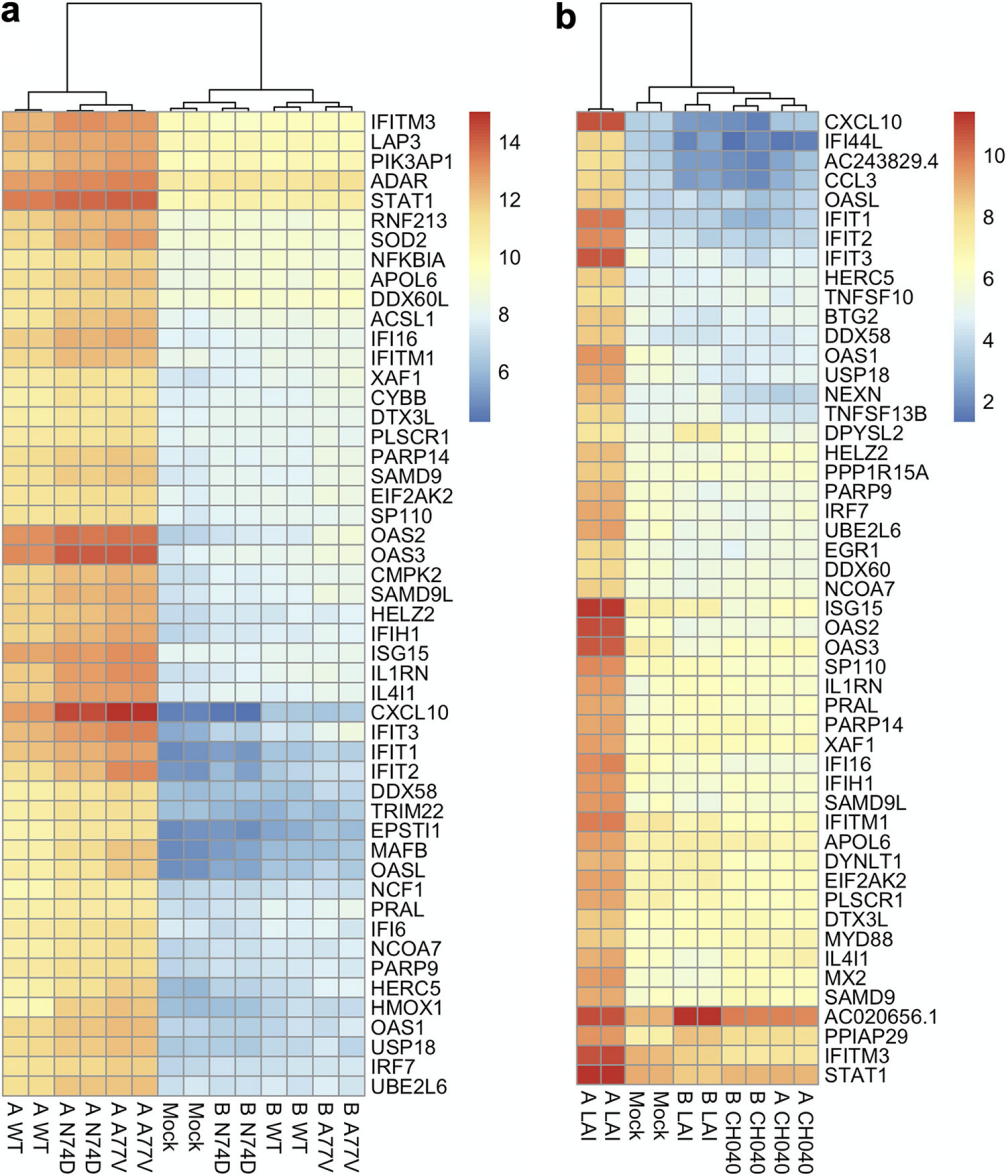
Role for the capsid in HIV-induced type I IFN responses. Effects of the viral capsid on HIV-induced type I IFN responses were examined using two CA mutant viruses (N74D and A77V) and an LAI-based chimeric virus encoding the CA subunit protein from the CH040 strain. All virus stocks were pseudotyped with the VSV-G protein and generated using two different methods (method A or B). (a and b) Infection of the two CA mutants (a) was performed in an experiment independent from the one for the CH040 chimeric virus (b). These heat maps were generated using genes that were significantly upregulated in duplicate samples of infection with the WT virus prepared with method A in each experiment. Specifically, genes with a log_2_ fold change value of greater than 2 were ranked based on adjusted *P* values. Then, the top 50 genes were selected to draw these heat maps, which were based on regularized log transformed data.

In the present work, we established experimental conditions in which, depending on a coexisting second signal, HIV-1 does or does not activate a type I IFN response. We used this experimental platform to reassess the role for the host factor CPSF6 in regulating HIV-1 DNA sensing. Previous work showed that the N74D mutant virus, which lost the ability to interact with CPSF6 (71) through the viral capsid, induced a type I IFN response in macrophages upon HIV-1 infection, whereas the WT virus did not (72). This finding was reproduced by Setiawan et al. (73) but not by others (74, 75). One advantage of our experimental system using THP-1 cells is that infectivity of the N74D virus is nearly equivalent to that of the WT virus (Fig. S4a), as opposed to macrophages in which the N74D virus is severely attenuated (74, 76). In our experimental system, the WT virus prepared using method B did not trigger a type I IFN response (Fig. 1 and 2). Thus, a prediction based on the previous observations was that infection with the N74D virus prepared using method B would result in activation of the type I IFN signaling pathway. However, this was not what we observed; the N74D mutant virus was similar to the WT virus in that they did not display transcriptome signatures indicative of activation of type I IFN signaling when prepared with method B (Fig. 8a). The A77V virus, another CPSF6 binding-deficient mutant (74), also failed to elicit induction of ISGs. We note that when viruses were prepared with method A, the two CA mutants appear to display slightly higher levels of upregulation of ISGs (Fig. 8a). Consistently, when type I IFNs were measured using a reporter cell line, levels of type IFNs induced by these CA mutants were marginally higher than those induced by the WT virus (Fig. S4b). However, these differences did not exceed 2-fold.

10.1128/mBio.02817-21.4 FIG S4 CPSF6-binding mutants do not overtly alter viral infectivity and the ability to induce type I IFNs in THP-1 cells. (a) Viral infectivity was plotted after infection of undifferentiated THP-1 cells with three viruses. The graph was generated by compiling the results of infection in two independent experiments. (b) Type I IFNs in the culture supernatant harvested at 1 dpi were quantified as described in the legend to Fig. 1. The graph was compiled from the results of six replicates of infection in two independent experiments. In all four graphs, each dot denotes the average, with error bars corresponding to the SEM. Statistical differences in IFN-I activity between the WT virus and CA mutant were examined using the two-tailed, unpaired Student’s *t* test. For the data sets of an inoculum of 60 μL, the WT virus differed from N74D (*P = *0.0171) and A77V (*P < *0.0001). For data sets of an inoculum of 30 μL, the WT virus differed from N74D (*P = *0.0005) and A77V (*P < *0.0001). Download FIG S4, TIF file, 0.7 MB. Copyright © 2021 Siddiqui and Yamashita. 2021 Siddiqui and Yamashita. https://creativecommons.org/licenses/by/4.0/ This content is distributed under the terms of the Creative Commons Attribution 4.0 International license.

A role for the viral capsid in regulating cGAS sensing of HIV-1 has been reported independently from CPSF6 (17, 52, 59, 60, 72). As described above, a virus strain possessing the capsid with elevated stability blocks type I IFN production upon HIV-1 when virus stocks were prepared using method A (Fig. 2). We validated this observation with the transcriptome approach; we found a drastically altered gene expression signature of cells infected with the virus harboring the capsid from the CH040 strain compared to the LAI virus. Specifically, when virus stocks were prepared using method A, the CH040 virus almost completely suppressed expression of many ISGs that were upregulated by the LAI virus (Fig. 8b). This observation supports the capsid’s critical role of serving as a shield against the innate immune recognition of viral DNA. Overall, these findings show that CPSF6 binding to HIV-1 capsid exerts very small, if any, effects on the outcome of cGAS-dependent sensing of HIV-1 in THP-1 cells.

## DISCUSSION

In this study, we leveraged an unbiased RNA sequencing approach for genome-wide gene expression profiling to unambiguously elucidate how intrinsic and extrinsic factors modulate innate sensing of HIV-1 DNA. A key conclusion is that we found a drastic effect of suboptimal levels of cGAS-independent innate signaling on the outcome of HIV-1 DNA sensing. HIV-1 infection by itself is not a potent stimulus that initiates cGAS-dependent IFN-I signaling. A second signal provided through activation of TLR pathways acts with HIV-1 reverse transcription to trigger a type I IFN response. Our results thus highlight how two signals in distinct innate immune pathways converge to amplify signaling outputs and thereby exert a profound impact on the outcome of HIV-1 DNA sensing by the host cGAS protein.

### Cross-talk between cGAS and TLR signaling pathways enables type I IFN induction upon HIV-1 infection.

In this work, we demonstrated that TLR signaling cooperates with the cGAS-STING pathway to enable HIV-1 infection to stimulate a type I IFN response in macrophages. Our findings are in line with previous work by Johnson et al., who demonstrated that TLR agonists R848 (TLR7/8 agonist) and PAM3 (TLR2 agonist) act together with the cGAS-STING pathway (51). Nonetheless, there are a few differences between their work and ours. One major difference is the timing of signaling events. In their work, HIV-1 DNA was needed to trigger cGAS activation first and then prime type I IFN responses by TLR ligands. When TLR ligands were added together with HIV-1 at the beginning of infection, they did not cause a synergistic effect on IFN production. On the contrary, in our hands, TLR agonists (LPS and HKLM) that were present at the time of infection cooperated with HIV-1 infection for type I IFN induction (Fig. 5 and 7). One potential way to account for this difference is cell-type dependency; Johnson et al. used monocyte-derived DCs as a target cell type (51), whereas our work used THP-1 and macrophages. Despite this difference, both experiments agree with the observation that robust IFN responses are mediated by cross talk between sensing pathways for TLR agonists and HIV-1 DNA. The cross talk between cGAS and TLR signaling pathways, which was observed in these reports, may be relevant to other pathogens (77). For instance, induction of type I IFNs by Neisseria gonorrhoeae requires both TLR4 and cGAS signaling pathways (78). Additionally, the coordination of signaling through TLR9 and cGAS pathways orchestrates production of type I IFN in response to infection with ectromelia virus (ECTV) (79).

How might TLR signaling potentiate IFN-I induction upon HIV-1 infection? Activation of TLR signaling pathways leads to expression of proinflammatory cytokines (Fig. 3). These proinflammatory cytokines may “prime” cells to allow IFN-I induction by HIV-1 infection. An alternative but not mutually exclusive model is a cooperative action at transcription of the *ifnb1* gene for IFN-β. LPS binding to the TLR4 receptor complex triggers two distinct pathways; one pathway activates NF-κB through the adaptor molecule MyD88, while the other activates IRF3 in a manner that depends on the adaptor molecule TRIF (2). Similarly, DNA sensing of cGAS activates both NF-κB and IRF3 (80). The enhancer of the human *ifnb1* gene contains binding sites for NF-κB and IRF3/IRF7 along with transcription factor ATF2/cJun (81). Recruitment and cooperative binding of these transcription factors to the enhancer lead to the assembly of an enhanceosome, a multiprotein complex that is required for transcriptional synergy and maximizes *ifnb1* gene expression (82). We speculate that cooperative binding of NF-κB and IRF3 to the enhancer, activated by both LPS and HIV-1 DNA, causes a high level of expression of the *ifnb1* gene, which culminates in a strong type I IFN gene signature, as represented by high levels of ISG expression. Previous work showed that cGAS expression was upregulated by LPS in A549 cells (83); however, our RNA-seq analysis did not show an increase in gene expression of the *cgas* gene (data not shown).

### HIV-1 infection *per se* is ineffective for activating cGAS-mediated IFN-I responses.

Our results showed that HIV-1 infection by itself (i.e., in the absence of an additional immunostimulatory molecule) did not lead to production of a detectable level of IFN-I (Fig. 4a). Macrophages displayed essentially the same phenotype, although higher inocula induced detectable levels of type I IFN (Fig. 1). Our transcriptome analysis also showed a lack of gene expression signatures that are characteristic of activation of IFN signaling by HIV-1 infection alone. However, expression of a small number of ISGs was increased upon HIV-1 infection by itself (i.e., using virus stocks prepared with method B), albeit to a limited extent (Fig. 2). These observations are in line with previous observations that a subset of ISGs, including viperin (also known as RSAD2), IFIT1, IFIT2, and IFIT3, was induced upon HIV-1 infection at an early phase of HIV-1, independently from both IFN-I induction and viral reverse transcription (13, 40, 43). Importantly, upregulation of these genes, most notably IFIT2 and IFIT3, was more pronounced in cells infected with method A virus than in those with method B virus (Fig. 3c). Thus, it is likely that LPS, a coexisting stimulus, contributes to this observation, although other immunomodulatory molecules (e.g., exosomes) may also be important (13). Nonetheless, a marked contrast in the breadth and magnitude of ISG expression between two different preparations of HIV-1 (Fig. 2) highlights a relative lack of HIV-1 by itself to cause cGAS-dependent IFN responses. Thus, overall, our data are consistent with some of the earlier reports showing that incoming HIV-1 particles are not a strong inducer for a type I IFN response that depends on cGAS sensing of HIV-1 DNA (11, 31, 42, 47, 60, 84–86). It should be noted, however, that the early phase is not the only period during which HIV-1 can be sensed, as multiple lines of evidence point to innate immune responses that are elicited by late events of the viral replication cycle (13, 15, 32, 33, 35, 51, 87, 88).

Several mechanisms can underlie the lack of a strong type I IFN response elicited by cGAS sensing of HIV-1 DNA (89). One possible mechanism is capsid-mediated shielding of viral DNA (54, 55). Multiple lines of evidence support a model in which intracellular virus complexes retain intact or near intact capsids in the cytoplasm and complete the disassembly process in the nucleus (90–93). Fittingly, PF74, a capsid-targeting inhibitor that promotes core opening, increases ISG expression by THP-1 cells infected with HIV-1 (59, 60), although this was not observed in CD4^+^ T cells (75). In addition, we found that stable cores (52) reduced levels of IFN-I production and prevented the upregulation of ISGs when HIV-1 infection was accompanied by a second immunostimulatory signal (Fig. 1 and 8).

Another mechanism involves cellular proteins that regulate DNA metabolism. Trex1, a cellular exonuclease, degrades aborted viral DNA (15, 31, 59), while SamHD1 may limit the amount of viral DNA to a minimum (94, 95). Finally, several accessory proteins encoded by HIV-1 and related lentiviruses are known to modulate an innate immune response through directly interacting with host molecules involved in various signaling pathways (85, 96–101) (reviewed in references 102 and 103). For instance, Vpr and Vpu have capabilities to dampen an innate immune response by downmodulating NF-κB activity (104–106). However, the relative contributions played by these accessory proteins in shaping the outcome of an innate immune response to HIV-1 remain somewhat uncertain, as conflicting findings have shown opposing activities (stimulatory versus suppressive) by these accessory genes (85, 101, 105, 107–110). Additionally, infection with virus devoid of these accessory proteins also results in a lack of type I IFN signaling activation in T cells (75).

### A cautionary tale.

We found that plasmid DNA prepared using a kit from Promega contained detectable levels of LPS. LPS was also present in virus stocks generated with LPS-containing plasmid DNA, suggesting that LPS was copurified with concentrated virus particles after ultracentrifugation with a sucrose cushion. This was somewhat unexpected because previous work showed that immunoreactive soluble factors from virus-producing cells were eliminated through a sucrose purification method (47). The amount of copurified LPS by itself was not sufficient for the gene expression signature for type I IFN signaling (Fig. 2) but was sufficient to have a drastic effect on the outcome of innate sensing of HIV-1 in THP-1 and macrophages. These observations are reminiscent of earlier work in which biological molecules copurified with virus stocks modulate the innate immune response. A residual amount of plasmid DNA used in virus production can be carried by a VSV-G-bearing tubulovesicular structure and elicit TLR9-dependent IFN-I release by pDCs (64). Similarly, genomic DNA packaged within lentiviral particles stimulated the cGAS pathway (111). DNA does not appear to be the only molecule that can be immunomodulatory, as extracellular vesicles along with their associated protein, HSP90α, were shown to induce an early increase in the expression of IFN-β and a number of ISGs (e.g., viperin and IFIT1) in MDMs (13). Notably, these observations are distinct from ours in the degree to which IFN-I production and upregulation of many ISGs were strictly dependent on HIV-1 DNA synthesis. Despite these differences, our work along with the papers discussed here will serve as a cautionary tale for future studies of the innate immune response to HIV-1 infection.

### Loss of CPSF6 binding does not render HIV-1 capable of stimulating type I IFN responses.

CPSF6, a host protein that binds to the capsid during postentry processes (71), was shown to help shield viral DNA from recognition by cGAS. CPSF6 knockdown (72) or a CA mutant deficient for CPSF6 binding (N74D) (72, 73) triggered elevated levels of IFN production (72, 73). A similar phenotype for the N74D mutant was not reproduced in other studies, which used different experimental settings (74, 75). In this study, we reassessed this matter by taking advantage of our experimental system using THP-1 cells, which offers a convenient platform, as the normalization of virus input is more straightforward in THP-1 cells than in primary macrophages. Specifically, N74D infectivity is severely impaired in primary macrophages prior to reverse transcription (76), a defect likely caused by restriction by TRIM34 (112). This property makes it difficult to normalize the virus input in macrophages, as the level of viral DNA would be different between the WT and N74D viruses. In contrast, infectivity of these viruses showed little or no difference in THP-1 cells (Fig. S4a). Given that the number of virions produced from transfected cells was not different between these two viruses, minimum normalization was needed for infection of THP-1 cells, which would rule out the possibility that differences in the number of virus-generated PAMPs and/or accompanying constituents influence the outcome of HIV-1 innate sensing.

Using this infection model of THP-1 cells, which are equipped with the functional cGAS sensing machinery, we found that neither the N74D nor the A77V virus enabled HIV-1 to induce a type I IFN response when the WT virus failed to do so (see gene expression profiles of virus stocks prepared with method B in Fig. 8a). We note that when virus stocks were prepared with method A, these CA mutants slightly increased the amount of soluble type I IFNs and expression levels of ISGs compared to the WT virus (Fig. 8a and Fig. S4a), although the magnitude of the difference was very small. No or very minor effects of CPSF6 binding mutations on the outcome of HIV-1 sensing are in marked contrast to the stable capsid of the CH040 strain, which almost completely prevents the induction of type I interferon responses to virus prepared with method A. Thus, these results do not appear to support a critical role played by CPSF6 for cGAS sensing of HIV-1 DNA in this experimental model using THP-1 cells. Viral capsids of these HIV-1 variants have been shown to uncoat at different kinetics; virus particles containing capsids of the CH040 strain exhibit a delayed rate of capsid disassembly (52), whereas those of CPSF6-deficient CA mutants uncoat at nuclear pores (90, 113). We speculate that the delayed uncoating of the CH040 strain allows its capsid to protect viral DNA from cGAS recognition. In contrast, a slight increase in ISG signals by CA mutants that block capsid interactions with CPSF6 may be explained by their uncoating kinetics that is distinct from that of the WT virus.

In summary, these results demonstrate that cGAS and TLR signaling pathways synergize to enable HIV-1 to induce a potent cGAS-dependent IFN response. Furthermore, the lack of a strong signature of IFN-I signaling supports the idea that HIV-1 by itself does not stimulate the cGAS sensing pathway.

## MATERIALS AND METHODS

### Chemicals.

The following molecules were purchased from InvivoGen: LPS derived from Escherichia coli 055 B5, HKLM (a freeze-dried, heat-killed preparation of Listeria monocytogenes), 2′3′-cGAMP produced in mammalian cells by cGAS, and polymyxin B produced by the soil bacterium Paenibacillus polymixa. Triton X-114 was purchased from Sigma-Aldrich. Nevirapine, raltegravir, and zidovudine (AZT) were obtained through the NIH AIDS Reagent Program, Division of AIDS, NIAID.

### Plasmid DNA.

Infectious molecular clones encoding GFP in place of the *nef* open reading frame are based on the LAI strain of HIV-1 (114). A clone that carries most of the Gag-encoding sequence from the CH040 strain as well as the one encoding the Env from the HIV-1 strain BaL have been described previously (52, 74). We also used molecular clones that carry HIV-1 CA mutations (N74D and A77V) (74). Plasmid DNA for expression of the VSV-G glycoprotein (pHCMV-G) and HIV-1 Gag-Pol (pCRV1-Gag-Pol) as well as DNAs for gene depletion vectors (pLKO.1-TRC control and pLKO.1-cGAS) have been described previously (52, 115, 116). pcDNA3.1-YU2 is an expression vector for Env from the HIV-1 strain YU2 (gift from Xueling Wu). Plasmid DNA was extracted using the PureYield Plasmid Midiprep system (Promega) or NucleoBond PC 10000 EF, Giga kit for endotoxin-free plasmid DNA (Macherey-Nagel).

### Cell culture.

Dulbecco’s modified Eagle’s medium (DMEM; Corning) supplemented with 10% fetal bovine serum (FBS; Sigma) and 1× penicillin-streptomycin (P/S; Corning) were used for maintaining human embryonic kidney 293T (HEK293T) cells. RPMI 1640 medium (Corning) supplemented with 10% FBS, 1× P/S, and 2 mM l-glutamine (Corning) were used for THP-1 cells. The HEK293-ISRE-Luc reporter cell line (gift from Xuguang Li) and C8166-CCR5 cell line (gift from J. Robinson) were described previously (53, 117). Peripheral blood mononuclear cells (PBMCs) were isolated from the whole blood obtained from anonymous donors (New York Blood Center) using a standard Ficoll-based density gradient centrifugation method. CD14^+^ monocytes were isolated from PBMCs using the EasySep human monocyte isolation kit (Stemcell). CD14^+^ monocytes were allowed to differentiate into MDMs for 6 to 8 days in RPMI 1640 supplemented with 10% FBS, 1× P/S, 2 mM l-glutamine, and 100 ng per mL of recombinant human granulocyte-macrophage colony-stimulating factor (GM-CSF; PeproTech) or recombinant human macrophage colony-stimulating factor (M-CSF; PeproTech).

### Viruses.

Virus stocks were generated by transient transfection of HEK293T cells using polyethylenimine (PolySciences). Virus stocks were produced with *env*-deleted molecular clones and pseudotyped with the VSV-G protein or HIV-1 Env of the YU2 strain by cotransfection with expression vectors for the respective envelope proteins. The Gag-Pol expression vector pCRV1-Gag-Pol along with the pHCMV-G plasmid were used to cotransfect with shRNA-encoding lentivirus vectors. For producing high-titer virus stocks, HEK293T cells were seeded in 100-mm plates at 5 × 10^6^ cells per plate. Culture medium was left untouched or replaced with fresh medium 1 day after transfection. Supernatant harvested at 2 days posttransfection was filtrated with a Steriflip filter unit (0.45 μm, polyvinylidene difluoride [PVDF]; Millipore) and gently layered onto 5 mL of 20% (weight per volume) sucrose solution in a polyallomer tube (Beckman Coulter). Ultracentrifugation was performed for 2 h at 24,000 rpm and 4°C using an SW28 rotor. Virus pellets were incubated with 3.2 mL of culture medium for at least 1 h at 4°C before suspension. Aliquots were prepared and stored at −80°C until use.

### Infection.

THP-1 cells were plated at 3 × 10^4^ cells per well in 96-well plates, unless otherwise stated. THP-1 cells differentiated with 50 ng per mL of phorbol 12-myristate 13-acetate (PMA) overnight were used in some experiments. MDMs were plated for infection at 5 × 10^5^ cells per mL in 96-well plates. THP-1 cells or MDMs were infected with various amounts of inoculum. Culture supernatant was harvested 1 day after infection for the IFN bioassay (see below). The number of GFP-positive cells was analyzed at 2 or 3 days after infection using the LSRII flow cytometer (BD Biosciences) or Guava easyCyte (Millipore).

### THP-1 transfection.

Transfection of THP-1 cells was performed using Lipofectamine 2000 reagent (Thermo Fisher Scientific). Transfection complexes were prepared in Opti-MEM (Gibco) using the required amount of each reagent, followed by mixing Lipofectamine at 0.5 μL per well. The complexes were incubated at room temperature for 5 min and then added to cells.

### Infection of THP-1 cells treated with LPS.

THP-1 cells treated with or without PMA were used to examine the effects of LPS on HIV-mediated induction of type I IFNs. Different amounts of LPS were either directly added to culture or transfected before viral infection as described above.

### Cotransfection of cGAMP and LPS.

THP-1 cells were seeded at 0.5 million cells per well in a 24-well plate. The cells were either left untreated or treated with PMA. Untreated cells were plated on the day of infection. Different LPS amounts were used to find that 2 μg per mL of LPS does not result in IFN induction.

### IFN bioassay.

Levels of IFN-I in the supernatant were measured using the reporter cell line HEK293-ISRE-Luc (53). This reporter cell line carries the IFN-stimulated response element (ISRE) within the promoter region, driving the expression of luciferase. HEK293-ISRE-Luc cells were plated on a 96-well flat-bottom plate at 3 × 10^4^ cells per well 1 day prior to use. One day after infection of THP-1 cells or MDMs, 50 μL of the culture supernatant was harvested and added onto the HEK293-ISRE-Luc cells. The cells were placed in a tissue culture incubator after the addition of supernatant. The next day, the cells were lysed with 20 μL of 1× buffer prepared from luciferase cell culture lysis 5× reagent (Promega) for 5 min at 37°C. Then, 5 μL of the lysates was transferred to an opaque 96-well plate, and 25 μL of the luciferase assay reagent (Promega) substrate was added. Luciferase signals were measured on a luminometer and shown as relative luciferase units.

### cGAS knockdown in THP-1 cells.

Expression of cGAS was reduced by shRNA transduction of THP-1 cells. Briefly, THP-1 cells plated at 1 million cells per well in 12-well plates were transduced with virus stocks that were concentrated by ultracentrifugation. Cells were also transduced with a virus stock generated using the parental vector. At 3 days after infection, the transduced cells were used for infection with WT HIV-1 or transfected with HT-DNA (2 μg per mL), cGAMP (20 μg/mL), or poly(I·C) (2 μg per mL). Culture supernatant from the treated THP-1 cells was used to measure IFN-I production using the HEK293-based reporter cell line. RNA was harvested from cells at 3 days after transduction and used for quantification of cGAS mRNA. Quantitative reverse transcription-PCR (qRT-PCR) was used to assess the knockdown efficiency of mRNA encoding cGAS. Extraction of total RNA and qRT-PCR were performed as described previously (52). The level of cGAS expression was determined using the 2^−ΔΔ^*^CT^* threshold cycle (*C_T_*) method and normalized to the mRNA level of the GAPDH (glyceraldehyde-3-phosphate dehydrogenase) gene.

### Endotoxin quantification.

The LAL chromogenic endotoxin quantification kit (Thermo Fisher Scientific) was used to measure the LPS content in DNA or virus stocks. Briefly, 50 μL of the endotoxin standard solution or samples to be tested was mixed with the LAL reagent in one well of a 96-well plate. The plate was covered and incubated at 37°C for 10 min. After incubation, 100 μL of the chromogenic substrate solution was added to each well, followed by incubation at 37°C for 6 min. The reaction was stopped by adding 100 μL of the stop reagent. Absorbance was read at 405 nm in a plate reader. A standard curve was generated using the absorbance values of standard samples. The formulated standard curve was used to determine the LPS (endotoxin) concentration for each sample. Endotoxin concentrations are shown as endotoxin units per mL (EU/ml).

### RNA sequencing and data analysis.

Total RNA was isolated from THP-1 cells using a commercial kit called Total RNA Isolation System (Macherey-Nagel). RNA samples were quantified on a spectrophotometer (NanoDrop ND-1000; Thermo Fisher Scientific) and quality-analyzed in a Qubit fluorometer (Thermo Fisher Scientific). The sequencing libraries were generated using a NEBNext Ultra II RNA library prep kit (New England Biolabs [NEB]) according to a protocol supplied by the manufacturer. Libraries were subjected to sequencing on a Hi-Seq 2500 platform at Genewiz, which generates 150-bp paired-end reads with a median depth of 4.7 million reads.

The quality of the obtained reads was assessed with the FastQC package (https://www.bioinformatics.babraham.ac.uk/projects/fastqc/). Low-quality bases with Phred quality scores of less than 25 were trimmed from either end using the software Cutadapt (118). The RNA-seq reads were then mapped to the human reference genome (GRCh38) using HISAT2 (119) and sorted using SAMtools (120). Mapped reads were counted with the featureCounts function in the Rsubread package (121) and used to generate count matrices. Differential expression analysis was performed based on raw read counts using DESeq2 (122). The analyzed genes were prefiltered by removing those with read counts pf less than 10 in total. A set of prefiltered genes was used to calculate log_2_ fold change (log_2_FC), *P* values, and adjusted *P* values. Significantly upregulated genes were selected with the following criteria: adjusted *P* values of <0.01, log_2_FC of  >2. Gene lists of significantly upregulated genes under certain conditions (e.g., WT LAI virus stock A versus mock) were subsequently used in GO enrichment analysis (123, 124). In the GO enrichment analysis, we focused on GO terms that belong to “biological process,” one of the ontology sources. GO terms that are overrepresented in a given gene list were identified using the enrichr software (125). These GO terms were ranked based on adjusted *P* values to identify the top 10 GO terms. Volcano plots and MA plots were drawn using the R packages EnhancedVolcano (https://github.com/kevinblighe/EnhancedVolcano) and ggpubr, respectively. For visualization of gene expression levels on heat maps, regularized log normalized counts were generated using DESeq2 and used to draw clustered heat maps using the R package pheatmap.

### Statistics.

Statistical analysis of data sets for IFN bioassays was performed using the two-tailed, unpaired Student’s *t* test or analysis of variance (ANOVA). The means of different conditions were compared using Tukey’s honest significance test. *P* values of less than 0.05 were considered statistically significant. ****, *P* < 0.0001; ***, *P* < 0.001; **, *P* < 0.01; *, *P* < 0.05. Analysis was performed with Prism software (GraphPad).

### Data availability.

The data set for the RNA-seq experiments was deposited in the NCBI Sequence Read Archive database (BioProject accession number PRJNA762357).
