# Supplementary figures and images for: Toll-Like Receptor (TLR) Signaling Enables Cyclic GMP-AMP Synthase (cGAS) Sensing of HIV-1 Infection in Macrophages
Source: mBio. 2021 Nov 30;12(6):e02817-21. doi: 10.1128/mBio.02817-21 (PMC8630538; doi:10.1128/mBio.02817-21)

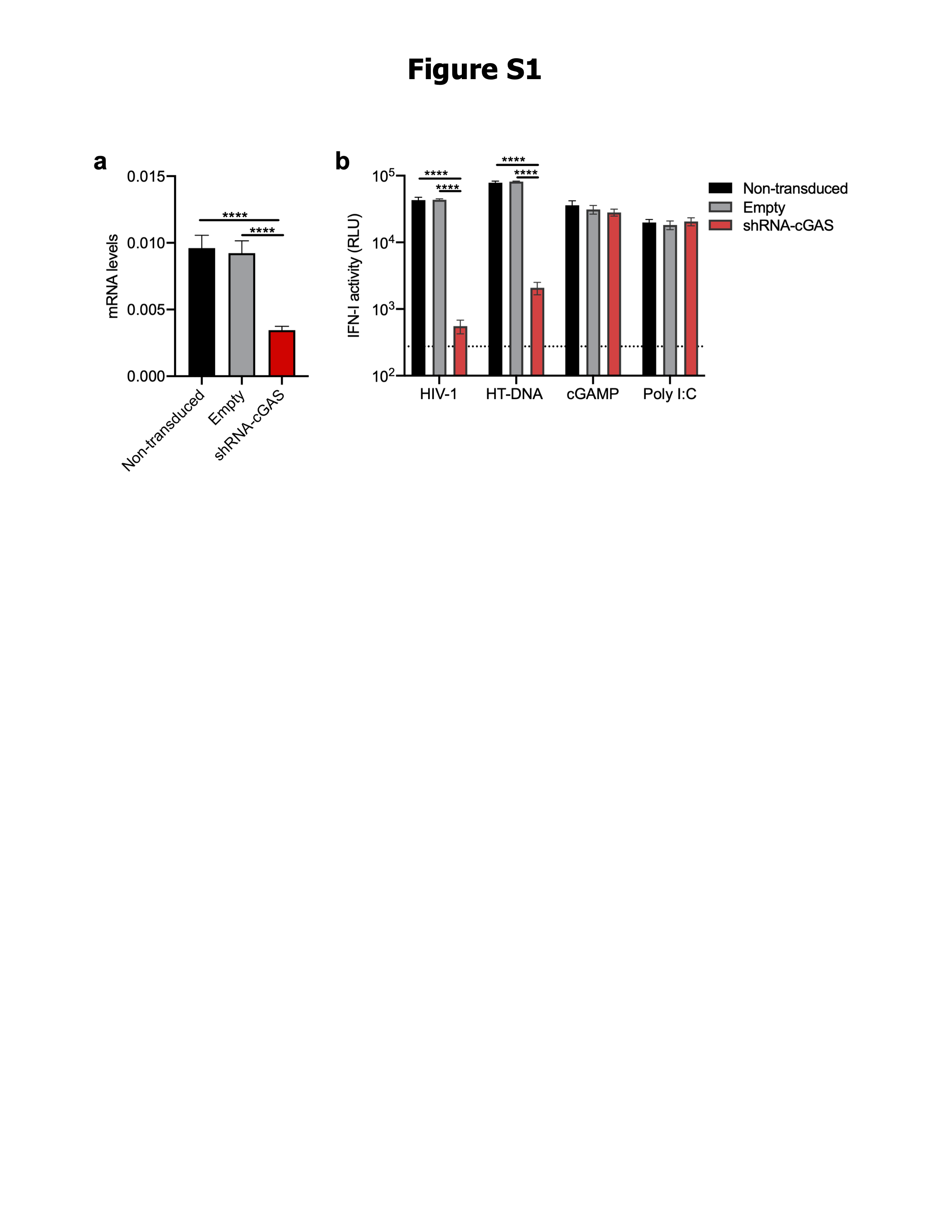

Supplement: FIG S1 [file mbio.02817-21-sf001.tif]

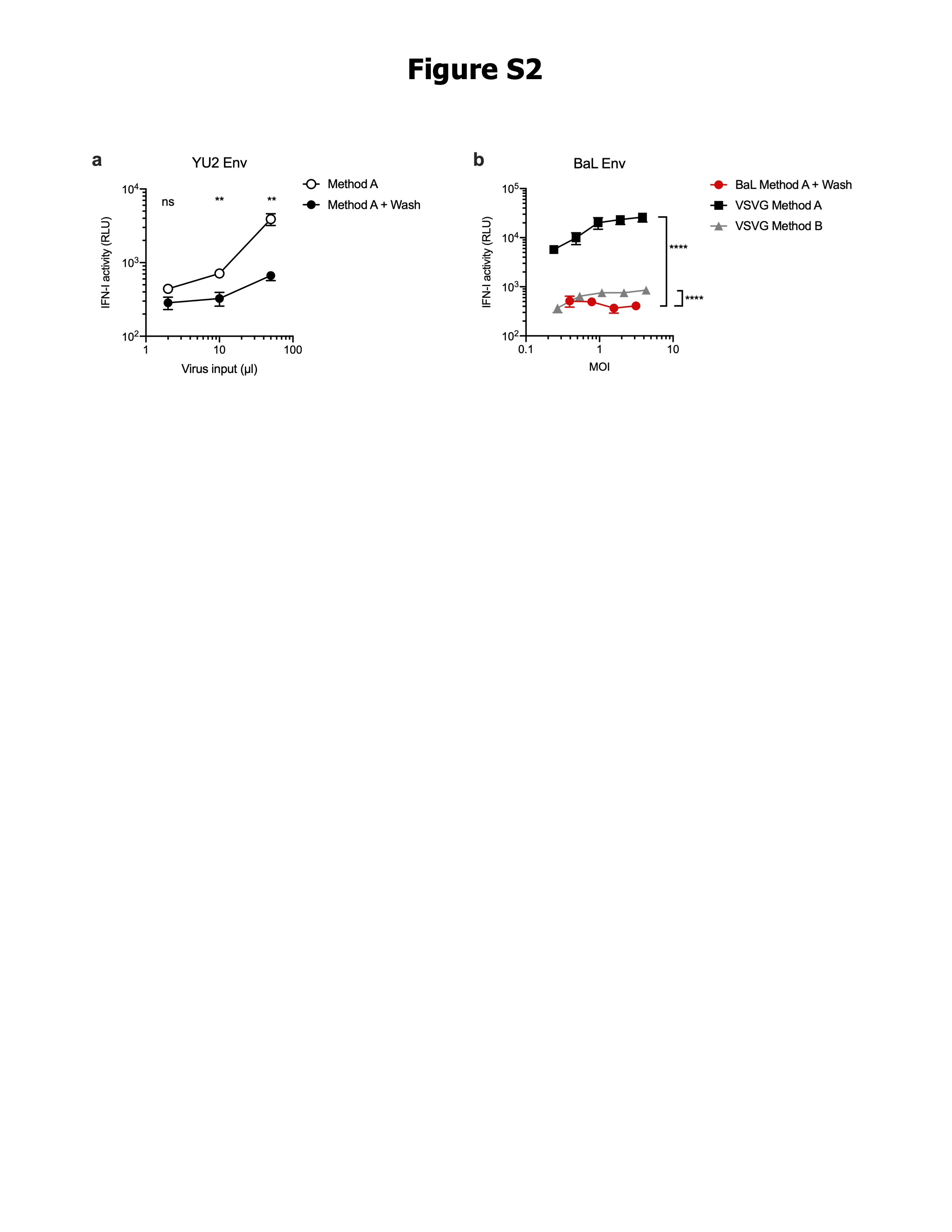

Supplement: FIG S2 [file mbio.02817-21-sf002.tif]

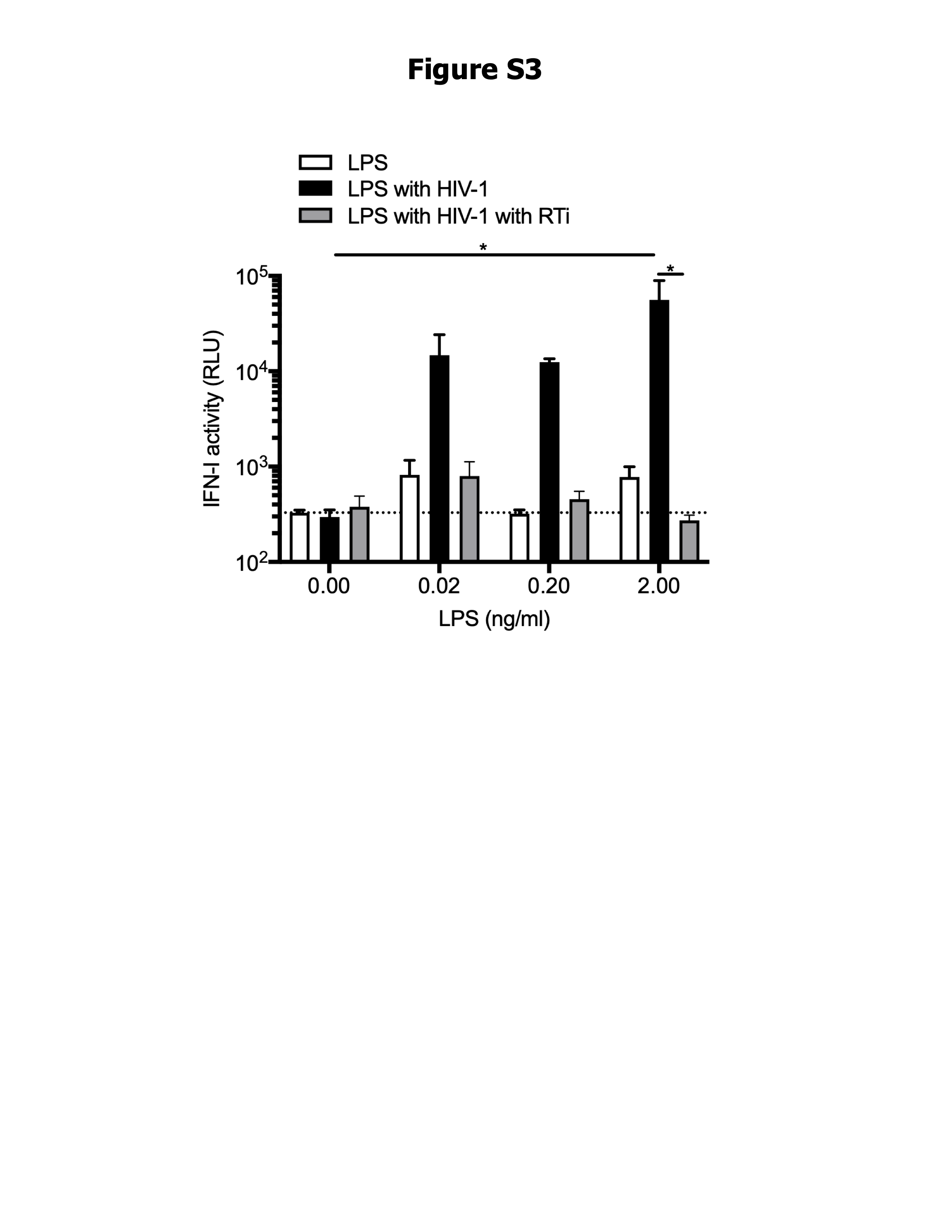

Supplement: FIG S3 [file mbio.02817-21-sf003.tif]

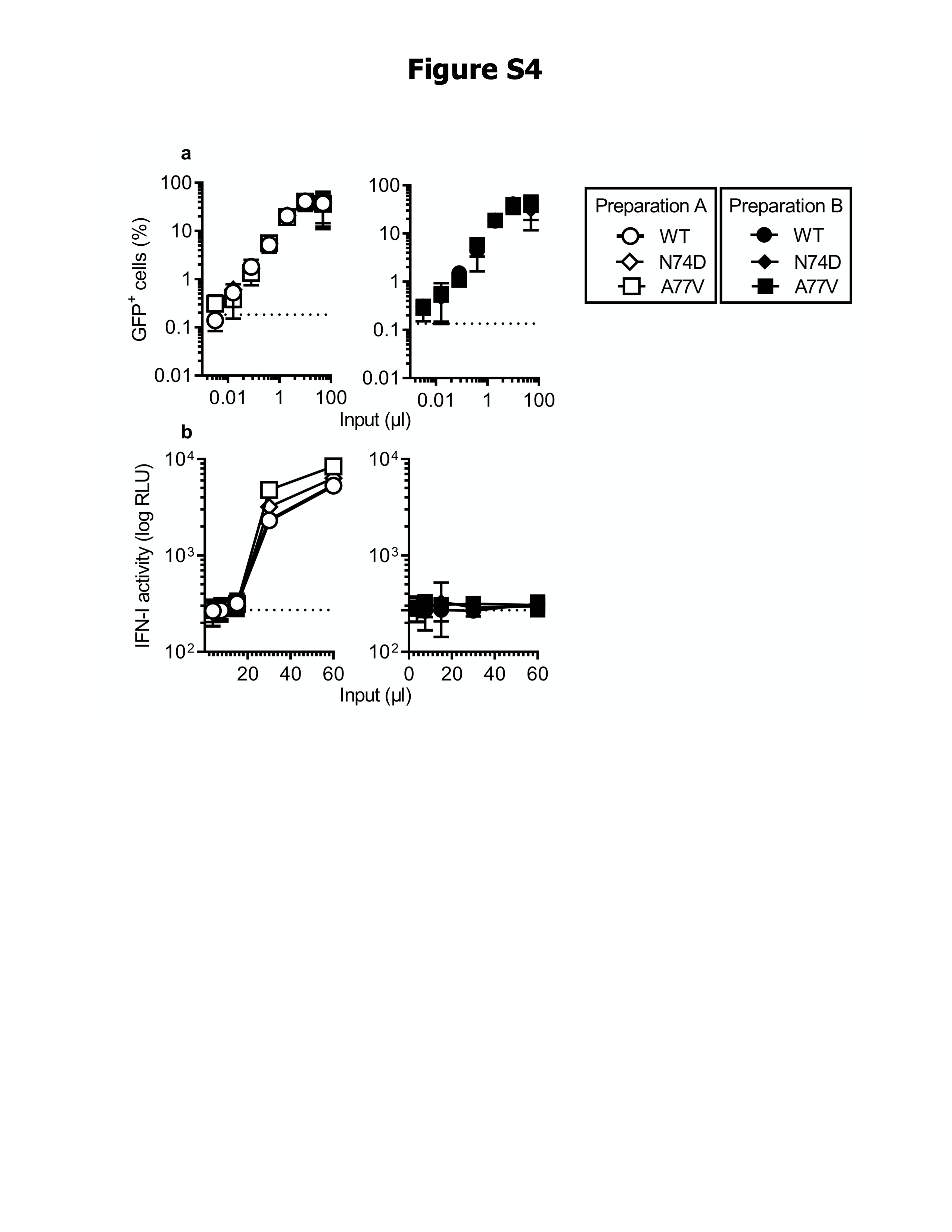

Supplement: FIG S4 [file mbio.02817-21-sf004.tif]
